# A80 SELF-COMPLETED QUESTIONNAIRES IDENTIFY SYMPTOMS IN A PROPORTION OF PATIENTS WITH CELIAC DISEASE CONSIDERED ASYMPTOMATIC DURING CLINICAL VISITS

**DOI:** 10.1093/jcag/gwad061.080

**Published:** 2024-02-14

**Authors:** N Chang, M Khaouli, D Armstrong, M Pinto-Sanchez

**Affiliations:** McMaster University, Hamilton, ON, Canada; McMaster University, Hamilton, ON, Canada; McMaster University, Hamilton, ON, Canada; McMaster University, Hamilton, ON, Canada

## Abstract

**Background:**

Patients with celiac disease (CeD) often experience symptoms despite adhering strictly to a gluten-free diet, and novel therapies are under development to help symptom management. However, recruitment for clinical trials [DA1] can be challenging, as screening is often based on symptoms reported to the physician during a clinic visit. However, symptoms reported by patients during clinic visits may not be an accurate reflection of the true extent of the symptoms.

**Aims:**

To assess whether gastrointestinal (GI) symptoms reported by patients to physicians during a clinic visit differ from those self-reported in questionnaires on the same day.

**Methods:**

A retrospective chart review of patients enrolled in the celiac registry (HiREB #5415, #16758) was performed. We included adult patients enrolled in our celiac registry from January 2021 to August 2023. We collected data on demographics such as age, sex, gender, time since CeD diagnosis (early diagnosis defined as within 2 years versus late diagnosis as greater than 2 years), CeD serology (TTG, DGP, EmA), in addition to GI symptoms 1) reported to the physician during the clinic visit, and 2) recorded on a validated questionnaire (Gastrointestinal Symptoms Rating Scale - GSRS) completed on the same day. A GSRS score greater than 30 for overall GI symptoms and a score greater than 2 for any individual symptom was considered symptomatic.

**Results:**

We reviewed a total of 174 patient charts that met the inclusion criteria during the defined time period. Overall, 107 patients reported symptoms during the clinic visit; of the remaining 67 patients who did not report symptoms at the clinic visit, 29 (43.2%) were identified as symptomatic by the GSRS. Compared to the clinical history, the GSRS identified higher rates of abdominal pain (77% vs 12.6%, p=0.0001), diarrhea (44.8% vs 11.4%, p=0.0001), bloating (87.3% vs 30.5%, p=0.0001), GERD (55.1% vs 5.1%, p=0.0001), constipation (57.4% vs 15.2%, p=0.0001) and nausea (56.3% vs 10.8, p=0.0001). There were no differences in responses between clinical visits and GSRS when subgrouped by gender, age, time since diagnosis or disease activity.

**Conclusions:**

Self-reported questionnaires identify a subgroup of patients with celiac disease who reported GI symptoms when completing a validated questionnaire despite reporting no symptoms during a clinic visit. Screening patients for clinical research studies is often based on symptoms reported to the physician during the clinical visit, and therefore this has direct implications for research recruitment in gastroenterology clinical trials.

**Funding Agencies:**

None

